# Inhibition of Histone Deacetylases Induces Cancer Cell Apoptosis Through the PERK Pathway of ER Stress Response

**DOI:** 10.1111/jcmm.70928

**Published:** 2025-10-29

**Authors:** Xuan Wang, Caiyun Yang, Yili Yang, Feng Xu, Donglan Yuan, Yuexi Gu

**Affiliations:** ^1^ China Regional Research Centre International Centre for Genetic Engineering and Biotechnology (ICGEB) Taizhou Jiangsu China; ^2^ Department of Critical Care Medicine The First People's Hospital of Changshu City, Changshu Hospital Affiliated to Soochow University Changshu China; ^3^ Department of Gynecology and Obstetrics Taizhou People's Hospital Affiliated to Nanjing Medical University Taizhou China

**Keywords:** apoptosis, ER stress, HDAC inhibitors, PERK pathway, quisinostat, unfolded protein response

## Abstract

Activation of the endoplasmic reticulum (ER) stress is an adaptive response to disturbed ER homeostasis caused by the accumulation of misfolded or unfolded proteins, or an acute increase in the entry of newly synthesised or mutated proteins into the ER lumen. Overwhelmed or prolonged ER stress causes apoptotic cell death or a maladaptive state of the cell, resulting in various pathological diseases including cancer, inflammation and aging. With a screening of a chemical compound library, here we show that inhibition of histone deacetylases (HDACs) induces ER stress, along with increased retro‐translocation of misfolded proteins from the ER lumen to the cytosol for proteasomal degradation. HDAC inhibitors (HDACi) activate the PERK‐eIF2α subbranch of the unfolded protein response (UPR), whereas the IRE1α and ATF6 pathways are not affected. Inhibition of the PERK subbranch with specific siRNA or a small molecule inhibitor ameliorates HDACi‐induced apoptotic cell death. In addition, a non‐phosphorylatable mutant of eIF2α, a critical substrate that transduces the PERK‐mediated ER stress response, abolishes apoptosis induced by HDACi, but not by the DNA damage reagent doxorubicin. HDACi reduce the sizes of tumours formed from wildtype but not eIF2α^S51A^‐mutant cells in a xenograft model, further demonstrating the involvement of the PERK subbranch in HDACi‐induced ER stress and cell death. Our study reveals novel effects of the well‐studied family of HDAC inhibitors, which can be explored further in clinics to treat certain types of cancer manifested with abnormal ER stress conditions.

## 1 Introduction

Histone deacetylases (HDACs) are a family of enzymes that remove acetyl groups from N(ε)‐acetylated lysine residues of both histone and non‐histone proteins [1, 2]. This modification allows histones to bind the DNA more tightly and thereby suppress gene expression in general. The deacetylation of non‐histone proteins, such as PTEN, HSP90, APE1/Ref‐1 and NF‐κB, also modulates many important cellular processes such as gene transcription and signal transduction [1, 3, 4]. It has been found that a variety of HDAC inhibitors (HDACi) are potent cytotoxic agents against tumour cells, and a number of them have been approved for the treatment of cancers, notably lymphomas [5, 6]. However, the spectrum of cancers responding to the therapeutic use of HDACi is rather limited, most likely due to their serious side effects and toxic effects [7]. Therefore, it is important to further understand how HDAC inhibitors induce cell growth and death, and to select inhibitors that are more effective for specific cancer cells.

In mammalian cells, most secretory and membrane proteins are targeted into the endoplasmic reticulum (ER) for folding and maturation [8]. When misfolded, abnormally modified or inappropriately expressed, proteins are accumulated in the ER and induce ER stress, which activates the unfolded protein response (UPR) that includes the PKR‐like ER kinase (PERK), inositol‐requiring enzyme 1α (IRE1α) and activating transcription factor 6 (ATF6) pathways [9, 10]. Their coordinated actions alleviate ER stress to maintain proteostasis, mainly through reducing protein syntheses, increasing protein‐folding capabilities, and promoting the degradation of misfolded proteins by a process named ER‐associated protein degradation (ERAD) [9, 11]. Of note, overwhelming ER stress and persistent activation of UPR can lead to the activation of cell death pathways to maintain homeostasis in tissues and organisms [12]. With the presence of hundreds of mutated proteins and deregulated growth, cancer cells undergo persistent ER stress and are presumably addicted to UPR for survival and growth [13]. Therefore, it has been proposed that cancer cells are susceptible to further ER stress or blocking of UPR, and targeting the ER stress process is a promising anti‐cancer strategy [14].

In the present study, we found that HDAC inhibitors markedly increased retro‐translocation of misfolded proteins, enhanced UPR, and induced cell death. Further experiments revealed that HDACi activate mainly the PERK‐eIF2α pathway of the UPR. In cells exposed to PERK inhibitor, or knocked out for PERK or harbouring the non‐phosphorylatable eIF2α S51A knock‐in mutation, HDACi‐induced cell death was significantly diminished, along with reduced induction of the UPR. These results indicate that HDAC inhibitors‐induced apoptosis of cancer cells requires the activation of UPR, particularly the PERK‐eIF2α subbranch. It is conceivable that further dissection of the death pathway will lay the foundation for precision treatment of various tumours with the ever‐increasing numbers of new HDAC inhibitors.

## 2 Materials and Methods

### 2.1 Cells

HEK293T (Cat. #GNHu44), HeLa (Cat. #TCHu187) and A549 (Cat. #TCHu150) cells were purchased from the Cell Bank of the Chinese Academy of Sciences (Shanghai, China). HeLa cells with eIF2α S51A mutation (eIF2α^S51A^) were generated by using homology‐directed recombination with CRISPR/Cas9 gene editing as described before [13]. HeLa cells engineered with the NanoBiT system for drug screening were kindly provided by Prof. Shengyun Fang (Department of Physiology, University of Maryland School of Medicine, Baltimore, USA). The system is composed of the Large BiT (LgBiT) subunit of the NanoLuc luciferase fused with the null Hong Kong (NHK) variant of α‐1‐antitrypsin and the Small BiT (SmBiT) subunit localised in the cytosol (the cells were designated as NHK‐HeLa cells in this study). All cells were maintained in Dulbecco's Modified Eagle Medium (DMEM, Thermo Fisher) supplemented with 10% fetal bovine serum (FBS) (Gibco, CA, USA) and 1% penicillin–streptomycin. Cells were cultured at 37°C in a humidified atmosphere containing 5% CO_2_.

To generate knockouts for PERK and ATF4, gRNA sequences were designed by using the online tool CRISPick (Broad Institute), and cloned into lentiCRISPRv2 for lentivirus packaging by transfection into HEK293T cells. After 72 h, the supernatants containing viruses were harvested and used to infect HeLa and A549 cells, followed by selection with puromycin at 0.8 μg/mL. The knockouts were validated by Western blotting using specific antibodies. Sequences of sgRNAs are: PERK, 5′‐TTAGCCAAGCTTGAACACCC‐3′; ATF4, 5′‐AGATGACCTTCTGACCACGT‐3′.

### 2.2 Antibodies and Reagents

Quisinostat (JNJ‐26481585), entinostat (MS‐275), belinostat, mocetinostat, abexinostat and GSK2656157 were purchased from Selleck Chemicals (Houston, USA). Doxorubicin, MG‐132, and Cell Counting Kit‐8 (CCK‐8) were obtained from MCE. LPS was purchased from Sigma‐Aldrich. The following antibodies used in this study were: anti‐eIF2A [EPR11042] (ab169528; RRID: AB_2819002), p‐eIF2α (ab235147; RRID: AB_732117) and p‐IRE1α (ab48187; RRID: AB_873899) from abcam; PERK (#5683; RRID: AB_10841299), p‐PERK (#3179; RRID: AB_2095853), ATF4 (#11815; RRID: AB_2616025), ATF6 (#65880; RRID: AB_2799696), PARP (#9542; RRID: AB_2160739), CHOP (#5554; RRID: AB_10694399), BiP (#3177; RRID: AB_2119845) and IκBα (#9242; RRID: AB_331623) were purchased from Cell Signalling Technology (CST). Mouse anti‐ubiquitin antibody (sc‐8017; RRID: AB_628423) was from Santa Cruz Biotechnology. β‐Actin (Cat#HC201) and GAPDH (Cat#HC301) antibodies were from TransGen Biotech (Beijing, China). HRP‐conjugated secondary antibodies were from Beyotime Biotechnology (Shanghai, China).

### 2.3 Cell‐Based Screening

Cell‐based screening was performed using the FDA‐Approved and Passed Phase I Drug Library purchased from Selleck Chemicals (Cat. #L3800). Compounds were dissolved in dimethyl sulfoxide (DMSO), and quisinostat and entinostat were prepared as stock solutions. The Promega NanoBit system was employed to measure luciferase activity as an indicator of protein retro‐translocation. NHK‐HeLa cells were seeded in 96‐well plates and treated with compounds at varying concentrations for 12 h. The luciferase activity was measured by using Nano‐Glo Live Cell Reagent (Promega, #N2012) according to the manufacturer's instructions.

### 2.4 Western Blotting

Cells were lysed using RIPA buffer (Beyotime) containing protease and phosphatase inhibitors (MCE) for 15 min on ice, followed by centrifugation at 13,000 rpm for 10 min to remove the cell debris. Protein concentrations were determined using the BCA Protein Assay Kit (Pierce). Cell extracts were mixed with 5× loading buffer and heated at 100°C for 10 min. Equal amounts of proteins were loaded onto an SDS‐PAGE gel for electrophoresis, followed by transfer to PVDF membranes for 2 h. Membranes were blocked with 5% non‐fat milk or bovine serum albumin (BSA) solved in Tris‐buffered saline with Tween‐20 (TBST) for 1 h at room temperature, followed by incubation with primary antibodies at 4°C overnight. After three washes with TBST, membranes were incubated with HRP‐conjugated secondary antibodies for 1 h at room temperature. After three washes with TBST, immunoreactive bands were visualised by chemiluminescence reagents (Pierce), and images were captured using the ChemicDoc gel documentation system. Densitometric analysis was performed using image analysis software.

### 2.5 Quantitative Real‐Time PCR (RT‐qPCR)

Total RNAs were extracted using RNAiso Plus (TaKaRa) according to the manufacturer's instructions. The concentrations and purity of RNA were assessed with a NanoPhotometer (IMPLEN). 500 ng total RNAs of each sample were used to synthesise cDNA using a PrimeScript RT reagent Kit (TaKaRa). The mRNA levels of BiP and CHOP were detected by real‐time PCR using the ABI StepOnePlus system (Applied Biosystems, Foster City, CA) and TB Green Premix (TaKaRa). GAPDH was used as an internal control. The following primers were used for amplification:

- BiP‐F: 5′‐ACCTATTCCTGCGTCGGTGT‐3′
- BiP‐R: 5′‐GCATCGAAGACCGTGTTCTC‐3′
- CHOP‐F: 5′‐GGAAACAGAGTGGTCATTCCC‐3′
- CHOP‐R: 5′‐CTGCTTGAGCCGTTCATTCTC‐3′
- GAPDH‐F: 5′‐GGTCACCAGGGCTGCTTTTA‐3′
- GAPDH‐R: 5′‐GAGGGATCTCGCTCCTGGA‐3′

### 2.6 Cell Viability Assay

Cell viabilities were determined using the Cell Counting Kit‐8 (CCK‐8) assay. Cells were plated in 96‐well plates at a density of 10,000 cells per well and treated with various concentrations of drugs including quisinostat for the indicated time points. Then each well was added with 10 μL CCK‐8 and incubated for 1 h at 37°C. The absorbance was measured with a microplate reader at 450 nm (ThermoFisher Scientific, USA).

### 2.7 Caspase‐3 Activity Assay

Caspase‐3 activity was measured using a Caspase‐3 Colorimetric Assay Kit (ab39401, abcam) according to the manufacturer's instructions. Briefly, cells were seeded in six‐well plates and treated with indicated concentrations of quisinostat for 12 h. Then the cells were lysed with lysis buffer provided in the kit. Reaction buffer containing the caspase‐3 substrate was added. They were mixed well in a 96‐well plate and incubated at 37°C for 1 h. The absorbance at 400 nm was recorded.

### 2.8 Xenograft Mouse Models

The animal study was approved by the Review Board of Animal Care and Use of Taizhou People's Hospital Affiliated to Nanjing Medical University. 6‐week‐old female athymic C57BL/6 nude mice were used for the xenograft model in this study. A total of 16 nude mice were randomly divided into four groups, two of which were injected with HeLa cells at the flank of the tail vein, while the other two groups received eIF2α S51A (knockin HeLa) cells. 4 days after inoculation of the cells, mice were started to be fed with Qui or drinking water as controls. The tumour volumes were measured every 3 days and growth curves were drawn until day 16. After the treatment period, the mice were euthanised, and tumours were excised, photographed, and weighed. Tumour volume was measured using the formula: volume = (length × width^2^)/2. Histopathological analysis of tumour tissues was performed by H&E staining. Further analysis was conducted by Western blotting to assess the expression levels of p‐eIF2α, cleaved PARP and CHOP.

### 2.9 Statistical Analysis

Statistical analyses were performed using SPSS statistical software and GraphPad Prism 6. Data were expressed as means ± standard deviation (SD) of at least three independent experiments. Statistical significance was determined using one‐way ANOVA tests, and *p*‐values less than 0.05 were considered significant.

## 3 Results

### 3.1 HDAC Inhibitors Enhance the Retro‐Translocation of Misfolded Proteins

**FIGURE 1:**
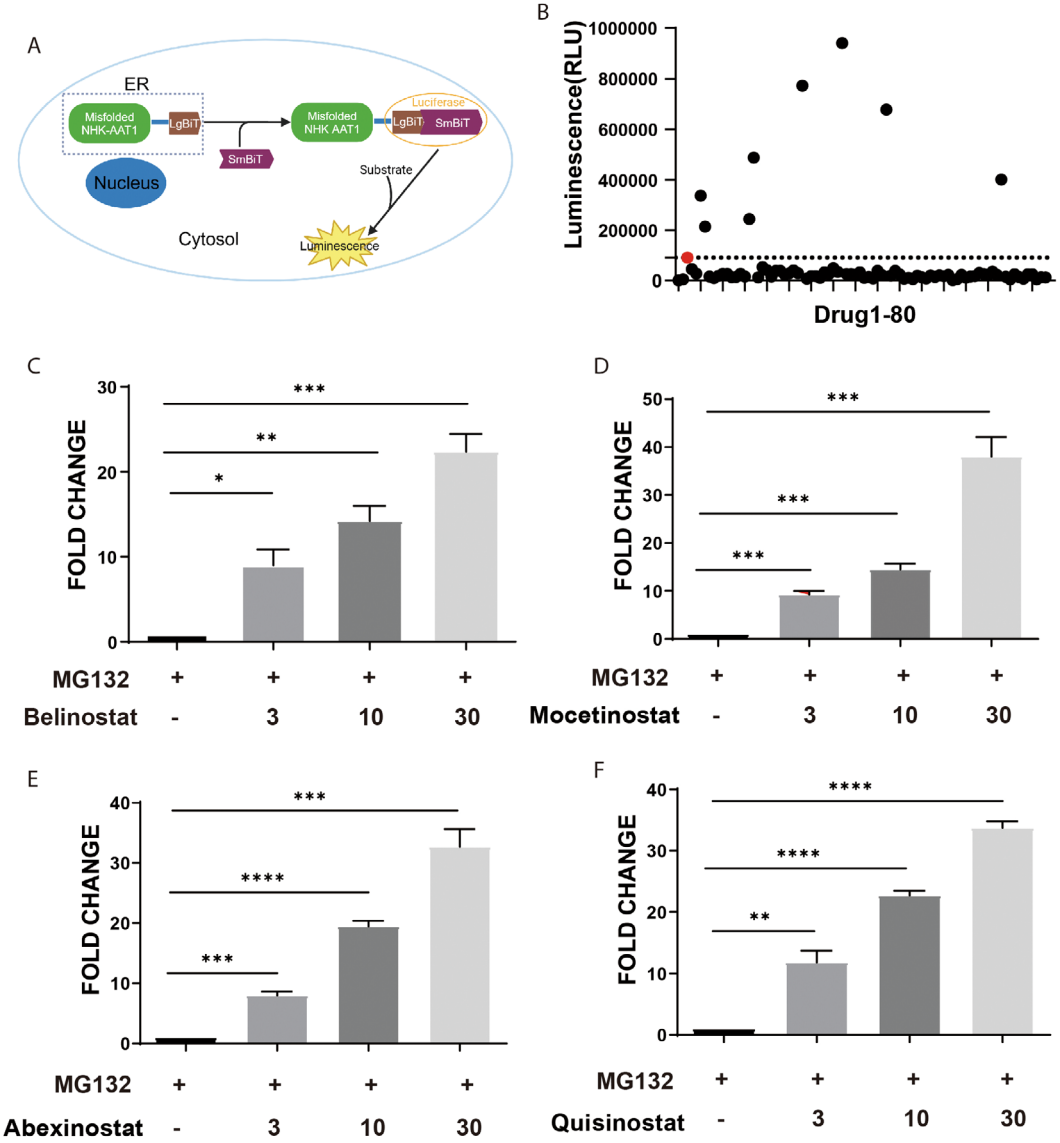
High‐throughput screening identifies HDAC inhibitors promoting retro‐translocation of misfolded proteins. (A) Schematic of the NanoBiT system used for the screening to identify chemical drugs that promote retro‐translocation of misfolded proteins into cytosol. (B) HeLa cells engineered with the NanoBiT system were subjected to a screen of drug library for 12 h. Luciferase activities were detected to identify drugs that can promote retro‐translocation of misfolded proteins. (C–F) HeLa cells engineered with the NanoBiT system were treated with different doses of HDAC inhibitors including belinostat (C), mocetinostat (D), abexinostat (E) and quisinostat (F) for 12 h, followed by MG132 for another 4 h. Luciferase activities were detected to validate protein retro‐translocation induced by HDAC inhibitors. *: *p* < 0.05; **: *p* < 0.01; ***: *p* < 0.001; ****: *p* < 0.0001 (one‐way ANOVA).

Unlike the secreted wild type protein, the null Hong Kong (NHK) variant of a‐1‐antitrypsin is retained within the ER due to its inability of proper folding, which can be transported to the cytoplasm for ubiquitination followed by ER‐associated protein degradation (ERAD) [15, 16]. As the transportation of proteins from ER lumen to cytoplasm (termed retro‐translocation) is a common gateway for all the proteins undergoing ERAD, it has been proposed as an attractive target for modulating protein fates in cells [17]. To monitor this process, we fused the large subunit (LgBiT, N‐terminal 1–156 amino acids) of the NanoLuc luciferase with the NHK variant, which is retained in the ER lumen. The small subunit (SmBiT, 11 amino acids) of the luciferase was expressed and localised in the cytoplasm of HeLa cells. Retro‐translocation of the NHK fusion protein into the cytoplasm results in the interaction of large and small subunits, thus generating luciferase activity that is enhanced in the presence of proteasome inhibitor, which can be measured in live cells (Figure 1A). The cells were then exposed to compounds from an FDA‐Approved and Passed Phase I Drug Library for 8 h, followed by the addition of MG132 for another 4 h. As shown in Figure 1B, 8 compounds increased significantly the luciferase activities, suggesting that they promoted the retro‐translocation of the NHK fusion proteins. Interestingly, the 8 compounds all belong to the family of HDAC inhibitors (HDACi). Further validations were performed with different inhibitors, including mocetinostat (relatively specific for HDAC1), abexinostat (relatively specific for HDAC1 and HDAC3), quisinostate (potent inhibitor of multiple HDACs 1–5 and 8), and belinostat (pan‐HDAC inhibitor). They all enhanced the luciferase activity dose‐dependently (Figure 1C–F).

Of note, blocking the degradation of the NHK‐luciferase may also elevate the luciferase activity. To rule out this possibility, we examined whether the HDAC inhibitor affects protein ubiquitination in cells. Unlike the proteasome inhibitor MG132, quisinostat treatment for 12 h did not raise the level of total protein ubiquitination in cells (Figure S1A). It also did not prevent LPS‐induced proteasomal degradation of IκBα (Figure S1B,C) [18]. Thus, HDACi apparently increases the luciferase activity by promoting protein retro‐translocation from the ER lumen to the cytosol without disrupting its proteasomal degradation.

### 3.2 HDACi Induces ER Stress and Activates the PERK‐eIF2α Pathway

**FIGURE 2:**
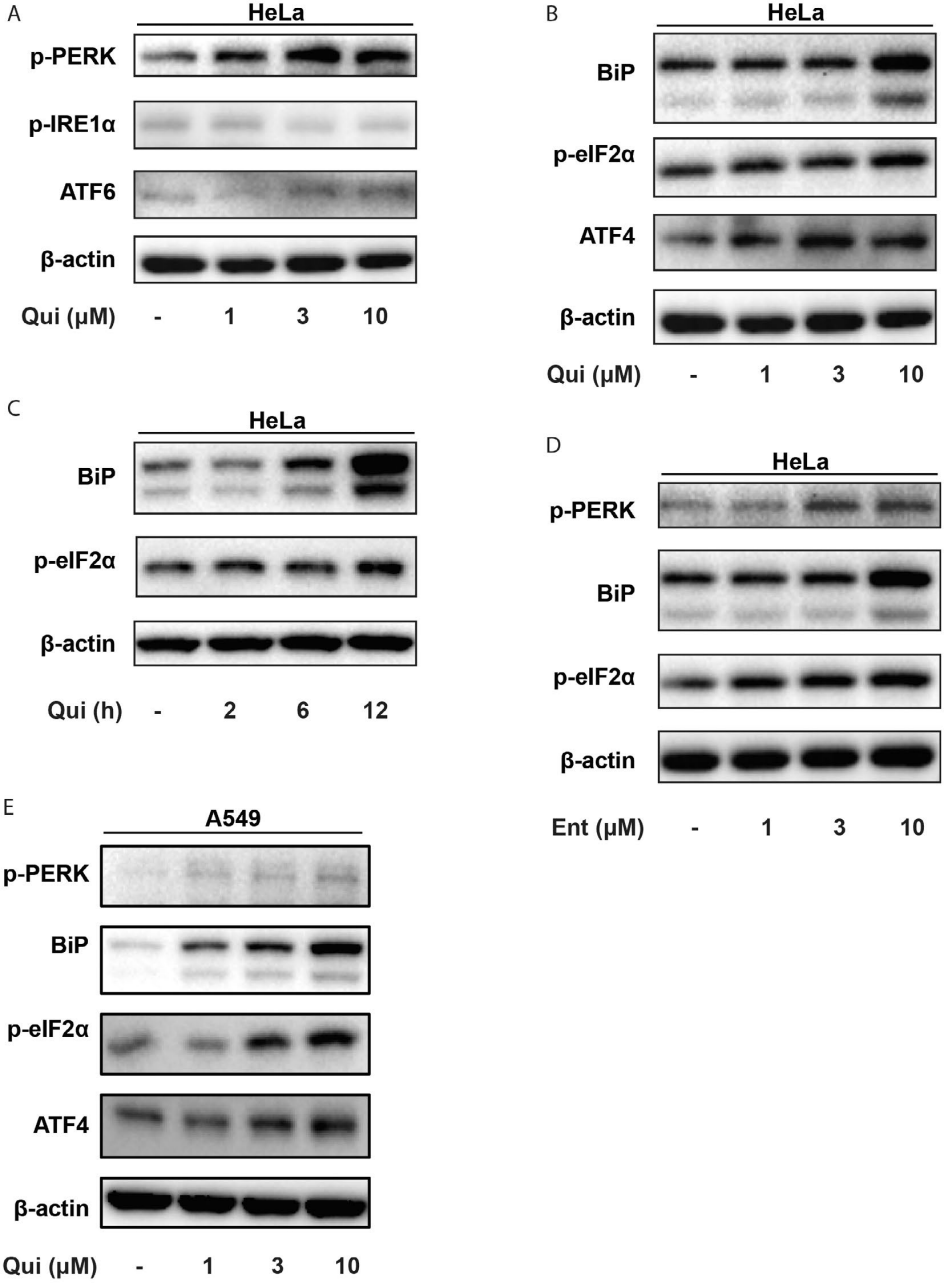
Quisinostat induces activation of the PERK subbranch of the ER stress response. (A) HeLa cells were treated with increased doses of quisinostat (Qui) for 6 h, followed by Western blot analyses of the activities of the ER stress pathways. (B, C) Qui induces activation of the PERK‐eIF2α subbranch of the ER stress response in dose (B) and time (C) dependent manners. (D) HeLa cells were treated with increased doses of the HDAC inhibitor entinostat for 6 h, followed by Western blot analyses for the activation of the PERK subbranch of ER stress pathways. (E) HDAC inhibition by quisinostat (Qui) activates the PERK‐eIF2α subbranch of the ER stress response in A549 cells.

To further understand how HDAC inhibition promotes the retro‐translocation of misfolded proteins, we examined the effects of HDACi on unfolded protein response (UPR) and its three main branches, namely PERK, ATF6, and IRE1α pathways. Quisinostat markedly elevated the level of BiP and activated PERK dose‐dependently in HeLa cells, whereas the statuses of ATF6 and IRE1α were not evidently altered (Figure 2A). Along the PERK pathway, quisinostat also induced a significant up‐regulation of eIF2α phosphorylation at Ser51, a critical substrate of PERK that inhibits global protein synthesis while selectively promotes stress‐related protein expression upon ER stress activation [19]. Furthermore, quisinostat treatment significantly induced the expression of transcription factor ATF4 that transduces signals of the PERK pathway in both dose‐ and time‐dependent manners (Figure 2B,C). We also investigated the impact of another HDAC inhibitor, entinostat (MS‐275). Similarly, entinostat also increased the levels of BiP, p‐PERK, and p‐eIF2α in a dose‐dependent manner (Figure 2D). Thus, HDAC inhibition causes significant ER stress and activation of the PERK pathway of UPR preferentially.

In order to validate the effects of HDAC inhibition on ER stress induction, we applied quisinostat on a non‐small cell lung cancer cell line A549, since aberrant expressions of HDACs are observed in lung cancer, and several HDAC inhibitors are tested in clinical trials for the treatment of lung cancer [20]. Similar to the results from HeLa cells, Qui induced the activation of PERK and eIF2α, as well as the up‐regulation of BiP and ATF4 in a dose‐dependent manner (Figure 2E). In contrast, there was almost no change in IRE1 phosphorylation or ATF6 expression level in response to increased concentrations of Qui, whereas thapsigargin (Tg) induced clear changes in IRE1 and ATF6 in A549 cells (Figure S1D). These results further demonstrate that HDAC inhibition by quisinostat activates the PERK–eIF2α subbranch of the ER stress response.

### 3.3 Inhibition of PERK Decreases Quisinostat‐Induced Effects on ER Stress and Apoptosis

**FIGURE 3:**
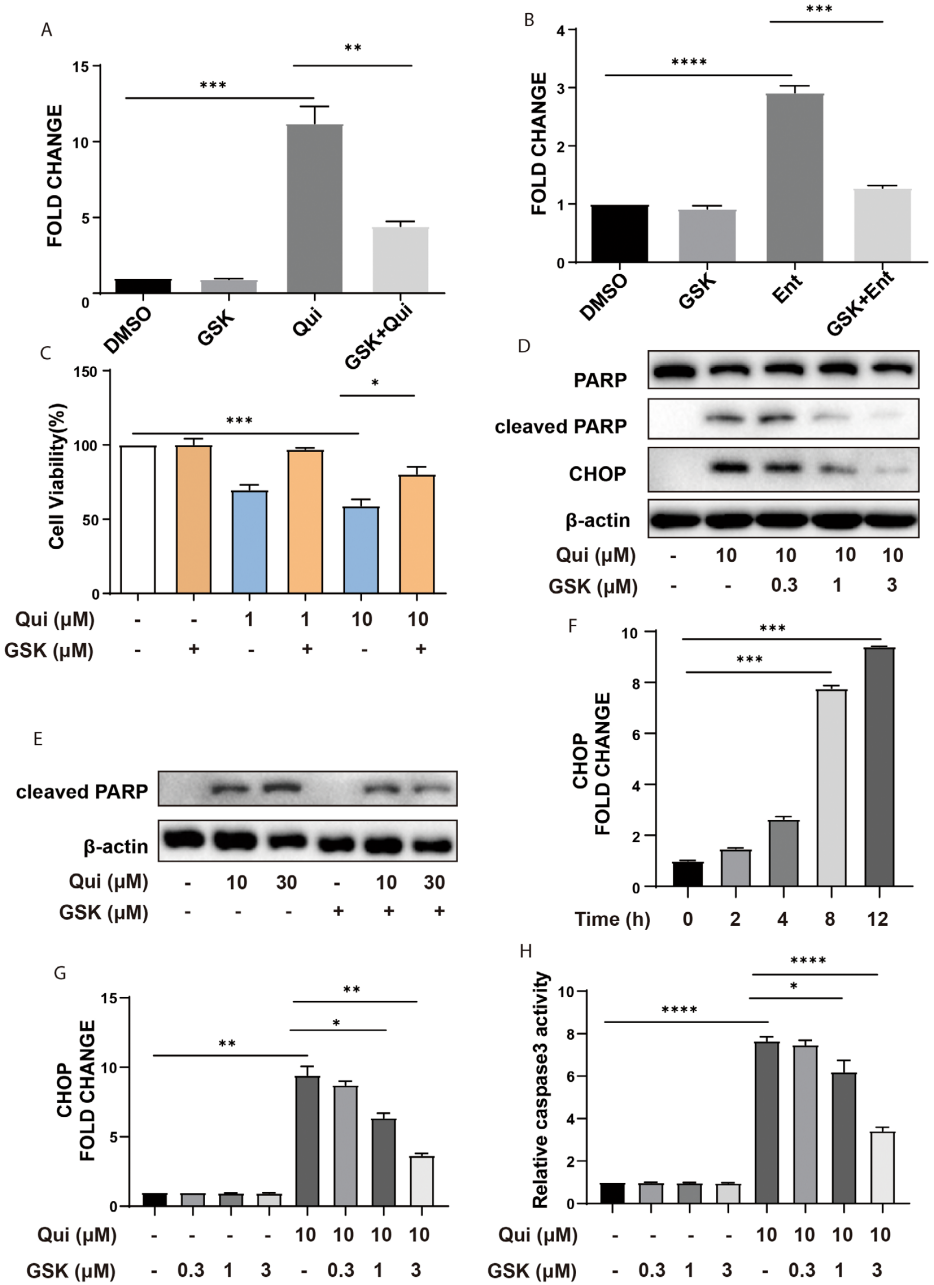
Inhibition of PERK attenuates HDACi‐induced ER stress and apoptosis. (A, B) NHK‐HeLa cells were treated with Qui (B) or Ent (C) for 12 h, followed by treatment with GSK2656157 (GSK) and subjected to luciferase activity assay to measure retro‐translocation of misfolded proteins. (C) HeLa cells were treated with quisinostat for 12 h, followed by treatment with PERK inhibitor GSK2656157 (GSK) for 1 h, and then subjected to cell viability analysis with CCK‐8 assay. (D, E) HeLa cells were treated with Qui and increased doses of GSK2656157 (D) or increased doses of Qui and GSK2656157 (E), and subjected to Western blot analyses of cleaved PARP and CHOP to measure apoptotic cell death. (F) HeLa cells were treated with Qui for different time points and subjected to RT‐qPCR analysis to measure the mRNA levels of CHOP. (G) HeLa cells were treated with Qui and increased doses of GSK2656157, and subjected to RT‐qPCR analysis to measure the mRNA levels of CHOP. (H) HeLa cells were treated with Qui and increased doses of GSK2656157, and subjected to Caspase‐3 activity assay to measure apoptotic cell death. *: *p* < 0.05; **: *p* < 0.01; ***: *p* < 0.001; ****: *p* < 0.0001. (one‐way ANOVA).

It has been shown that UPR helps to maintain proteostasis in the ER through decreasing protein synthesis, increasing protein folding capabilities, and enhancing misfolded protein degradation [21]. We found recently that attenuation of protein translocation into the ER lumen is an additional protective measure to mitigate ER stress [13]. However, overwhelmed and persistent ER stress may also activate the cell death programme. We asked whether activation of PERK is required for the cytotoxic effects of HDAC inhibition by using a PERK inhibitor, GSK2656157. As shown in Figure 3A,B, both quisinostat and entinostat‐induced increases in luciferase activity in the HeLa cells were largely suppressed by the PERK inhibitor. Meanwhile, quisinostat induced a marked decrease in cell viability as measured with CCK‐8, which was ameliorated dose‐dependently by GSK2656157 (Figure 3C). Further, quisinostat treatment also led to the cleavage of PARP, an indicator of apoptotic cell death, which was also significantly reduced by the PERK inhibitor (Figure 3D).

It has been shown that ER stress may activate the cell death programme through Regulated IRE1α‐dependent decay (RIDD) of mRNAs, caspase 12 [22], and PERK‐dependent induction of C/EBP homologous protein (CHOP), which functions as a dominant‐negative inhibitor by forming heterodimers with other C/EBP members, such as C/EBPα, to promote apoptosis [23]. As HDACi acts mainly on the PERK pathway, we examined the expression of CHOP in the absence or presence of GSK2656157. The levels of CHOP mRNA elevated gradually upon exposure to quisinostat (Figure 3E) and were largely prevented by the PERK inhibitor treatment dose‐dependently (Figure 3F,G). Along with the down‐regulation of CHOP mRNA, caspase 3 activity in the cells was also reduced markedly (Figure 3H). Taken together, these data indicate that apoptosis induced by HDACi is likely mediated by the PERK‐eIF2α‐ATF4‐CHOP axis of the UPR.

To further delineate the roles of the PERK‐eIF2α‐ATF4 axis in Qui‐induced apoptosis mediated by the ER stress response, we generated PERK and ATF4‐knockout cells in A549 and tested their responses to Qui. Qui‐induced apoptosis was significantly inhibited in both PERK and ATF4 knockout A549 cells, consistent with that observed in HeLa cells, where inhibition of PERK with a specific compound attenuated apoptotic cell death (Figure S2A,B). Knockout of PERK also prevented Qui‐induced eIF2α phosphorylation as well as ATF4 and CHOP up‐regulation, consistent with a role of PERK at the upstream and CHOP as a pro‐apoptotic factor under ER stress conditions [23].

### 3.4 Phosphorylation of eIF2α Is Required for Quisinostat‐Induced Apoptosis

**FIGURE 4:**
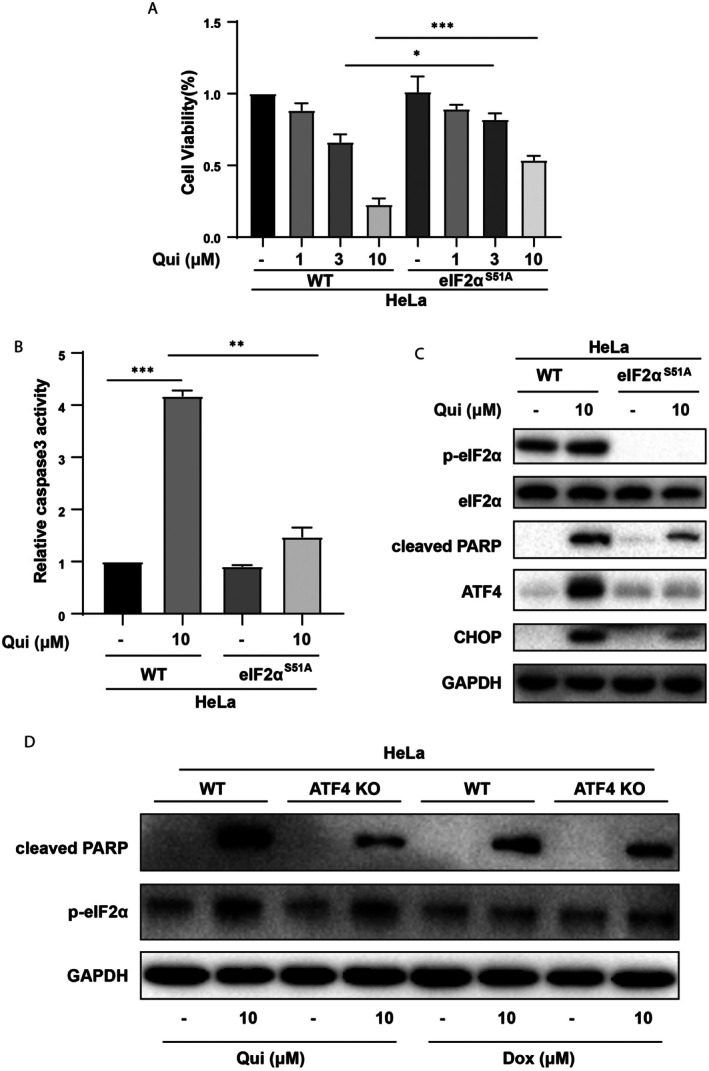
Non‐phosphorylatable mutation of eIF2α^S51A^ inhibits quisinostat‐induced ER stress and apoptosis. (A) Wildtype and eIF2α^S51A^‐mutant HeLa cells were treated with increased doses of Qui for 3 days and subjected to CCK‐8 to measure cell viabilities in response to HDAC inhibition. (B) Wildtype and eIF2α^S51A^‐mutant HeLa cells were treated with Qui for 24 h and subjected to Caspase‐3 activity assay to measure apoptotic cell death. (C) Wildtype and eIF2α^S51A^‐mutant HeLa cells were treated with Qui for 24 h and subjected to Western blot analyses of cleaved PARP, ATF4 and CHOP expressions, as well as eIF2α phosphorylation at Ser51. (D) Wildtype and ATF4‐knockout HeLa cells were treated with Qui or Dox for 24 h and subjected to Western blot analyses to show the induction of apoptosis and ER stress. *: *p* < 0.05; **: *p* < 0.01; ***: *p* < 0.001. (one‐way ANOVA).

To further elucidate the involvement of the PERK‐eIF2α axis in HDACi‐induced cell death, we made use of HeLa cells harbouring an eIF2α with the serine 51 residue mutated into a non‐phosphorylatable alanine (eIF2α^S51A^). Compared with parental wild‐type HeLa cells, the eIF2α^S51A^‐mutated cells were more resistant to the cytotoxic effects of quisinostat as assessed by CCK‐8 and caspase 3 activity assays, as well as PARP cleavage (Figure 4A–C). In addition, HDACi‐induced ATF4 and CHOP up‐regulation were almost diminished in cells with the S51A mutation (Figure 4D), consistent with the requirement of eIF2α phosphorylation by PERK in the selective induction of these proteins. In contrast, HeLa cells with wild‐type eIF2α and the S51A mutation showed similar levels of PARP cleavage and reduction of viabilities when exposed to the chemotherapeutic agent doxorubicin (Figure S2C), which is known for inducing DNA damage and activating the p53 pathway, but not inducing BiP. Taken together, these data indicate that HDACi selectively triggers ER stress and the PERK‐eIF2α pathway to generate cytotoxic effects.

### 3.5 The PERK‐eIF2α Axis Mediates Quisinostat‐Induced Apoptosis of Tumour Cells in a Xenograft Model

**FIGURE 5:**
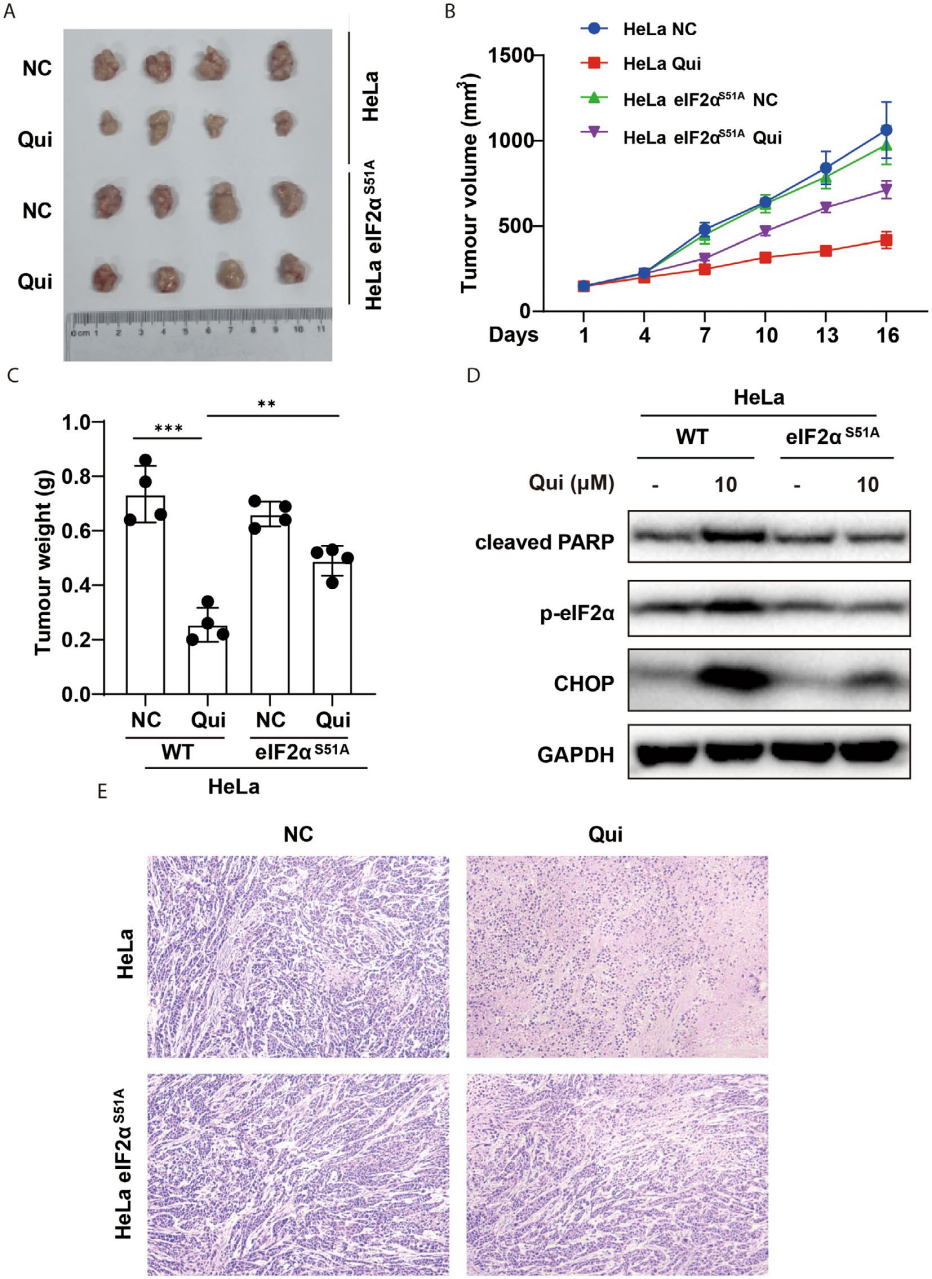
Quisinostat‐induced tumour cell death in vitro is dependent on the phosphorylation of eIF2α. (A) Wildtype or eIF2α^S51A^‐mutant HeLa cells were injected into nude mice to form tumours in a xenograft model and treated with or without quisinostat for 16 days. Qui treatment significantly reduces the tumour sizes from wildtype cells, but not that from eIF2α^S51A^‐mutant HeLa cells. (B, C) Quantifications of the sizes (B) or weights (C) of tumours treated with or without Qui as in (A). (D) Western blot analyses of PARP cleavage and ER stress induction in tumours formed from wildtype or eIF2α^S51A^‐mutant HeLa cells. (E) Histological staining shows that Qui induced significant tumour cell death formed from wildtype but not eIF2α^S51A^‐mutant HeLa cells. **: *p* < 0.01; ***: *p* < 0.001.

To test whether the ER stress‐mediated pro‐apoptotic effects of Qui can be applied for the treatment of cancer in vivo, we generated a xenograft model by injecting wildtype and eIF2α^S51A^‐mutated HeLa cells in nude mice to form tumours, followed by feeding the mice with or without quisinostat. As shown in Figure 5A,B, wildtype and eIF2α^S51A^‐mutated HeLa cells formed tumours with similar sizes. However, while treatment with Qui significantly shrank the sizes and weights of tumours formed by wildtype cells, the same treatment only caused a mild reduction of the tumours formed from eIF2α^S51A^‐mutated cells (Figure 5A–C). Further analyses showed that this is due to a strong resistance to Qui treatment after the non‐phosphorylatable eIF2α S51A mutation (Figure 5D). Histological staining of the tumours also showed extensive cell debris and apoptotic bodies in wildtype but not eIF2α^S51A^‐mutated tumour cells (Figure 5E), further demonstrating that the eIF2α S51A mutation confers resistance to quisinostat. Taken together, these results further validate that the apoptotic cell death induced by HDAC inhibition requires the PERK‐eIF2α axis of the ER stress response.

## 4 Discussion

The processes of acetylation and deacetylation of histones by histone acetyltransferases (HATs) and histone deacetylases (HDACs) have profound effects on gene transcription and expression [24, 25]. For example, it was found that inhibition of HDACs in cancer cells slowed down replication forks, activated dormant origins, and induced DNA damage [26, 27]. It is generally believed that HDAC inhibitors regulate the balance of pro‐apoptotic and anti‐apoptotic genes, leading to the activation of both extrinsic and/or intrinsic apoptotic pathways [28]. Given the large number of HDACs in mammalian cells, it is still not well understood how the altered gene expression induced by a particular HDAC inhibitor is transduced into the specific signal that activates the cell death programme [29]. In the present study, we found that HDACi turn on the PERK‐eIF2a pathway effectively, which induces the expression of CHOP, a mediator of UPR‐mediated apoptosis. Further, both blocking the PERK pathway by a small molecular inhibitor and preventing the phosphorylation of eIF2a through knock‐in mutation diminished apoptosis in response to HDACi. Thus, it is evident that the cytotoxic action of HDAC inhibitors requires activation of the PERK‐eIF2a pathway.

ER stress may result from endogenous abnormalities, including misfolded protein accumulation, inappropriate protein expression, and calcium fluctuation, as well as exogenous stimuli, such as nutrient deprivation and hypoxia [30, 31]. It has been reported by a number of studies that HDACi may induce ER stress in different cells [32, 33]. Since HDAC inhibitors induce dramatic changes in protein expression, it is plausible to hypothesise that the acute increases of protein levels upon HDAC inhibition are likely responsible for their effects on ER stress induction. Interestingly, a recent study showed that HDACi is able to reactivate provirus expression in T cells [34]. Expression of BiP/GRP78, an important component of UPR, was also increased by HDAC inhibitors, which may reflect a feedback mechanism for cells to cope with the induced ER stress. Of note, alteration of non‐histone protein acetylation by HDACi may also lead to ER stress [35]. In addition, it has been reported that HDAC inhibitors can directly affect DNA replication and cause DNA damage that may result in ER stress [26]. Thus, it is conceivable that multiple mechanisms may contribute to the initiation of ER stress in different cells after being exposed to HDACi.

HDACs are often highly expressed in tumour cells, and HDAC inhibitors are potent cytotoxic agents that induce apoptosis in a variety of cancers [36]. However, this type of therapeutics has only been approved for treating haematopoietic malignancies, likely due to their severe toxic and side effects in solid cancers [7, 37]. Our finding that HDACi induce cell death through the PERK‐eIF2a pathway suggests that the variation of the UPR status in tumour cells may have profound effects on the actions of HDACs. Therefore, it is conceivable that we should test the therapeutic effects of HDACi by concomitant examination of the basal ER stress activities. Of note, HDACi may have additional influence on various cancer cells. It was found that kaempferol, a natural flavonoid compound, is able to inhibit HDAC, trigger ER stress, and induce autophagic cell death via the IRE1‐JNK‐CHOP axis [38]. Interestingly, ER stress induced by HDACi may also affect antigen presentation and cytokine secretion, which enhance anti‐tumour immunity effectively [35]. Thus, the ER stress linkage of HDAC inhibition likely represents a novel avenue for exploring its therapeutic application under a variety of circumstances.

## Author Contributions

**Yuexi Gu:** conceptualization (lead), formal analysis (equal), funding acquisition (supporting), investigation (equal), methodology (equal), supervision (equal), writing – original draft (equal), writing – review and editing (equal). **Xuan Wang:** data curation (equal), methodology (equal), validation (equal), visualization (equal), writing – original draft (equal). **Caiyun Yang:** data curation (equal), formal analysis (equal), methodology (equal), validation (equal), writing – original draft (equal). **Yili Yang:** data curation (equal), formal analysis (equal), funding acquisition (equal), resources (equal), supervision (equal), writing – review and editing (equal). **Feng Xu:** formal analysis (equal), methodology (equal), project administration (equal), resources (equal), validation (equal), writing – original draft (equal). **Donglan Yuan:** conceptualization (equal), funding acquisition (equal), investigation (equal), project administration (equal), resources (equal), supervision (equal), validation (equal), writing – review and editing (equal).

## Conflicts of Interest

The authors declare no conflicts of interest.

## Supporting information


**Figure S1:** The HDAC inhibitor quisinostat does not inhibit proteasomal activity. (A) NHK‐HeLa cells were treated with quisinostat (Qui) or MG132, and subjected to Western blot analysis for ubiquitination. (B‐C) NHK‐HeLa cells were treated with quisinostat and/or MG132, followed by LPS stimulation and subjected to Western blot analysis of IκBα degradation. (D) Increased concentrations of HDAC inhibitor quisinostat (Qui) does not activate IRE1α or AFT6 pathways of the ER stress response.

**Figure S2:** The PERK‐eIF2α pathway is required for apoptosis induced by HDACi but not by doxorubicin (A) Knockout of PERK in A549 cells inhibits Qui‐induced apoptosis and p‐eIF2α, ATF4 and CHOP protein levels. (B) Knockout of ATF4 in A549 cells inhibits Qui‐induced apoptotic cell death. (C) Wildtype and eIF2α^S51A^‐mutant HeLa cells were treated with doxorubicin (Dox) for 24 h and subjected to Western blot analyses of cleaved PARP and BiP expressions, as well as eIF2α phosphorylation at Ser51.

## Data Availability

The data that support the findings of this study are available from the corresponding author upon reasonable request.
