# Supplementary figures and images for: Inhibition of Histone Deacetylases Induces Cancer Cell Apoptosis Through the PERK Pathway of ER Stress Response
Source: J Cell Mol Med. 2025 Oct 29;29(21):e70928. doi: 10.1111/jcmm.70928 (PMC12571189; doi:10.1111/jcmm.70928)

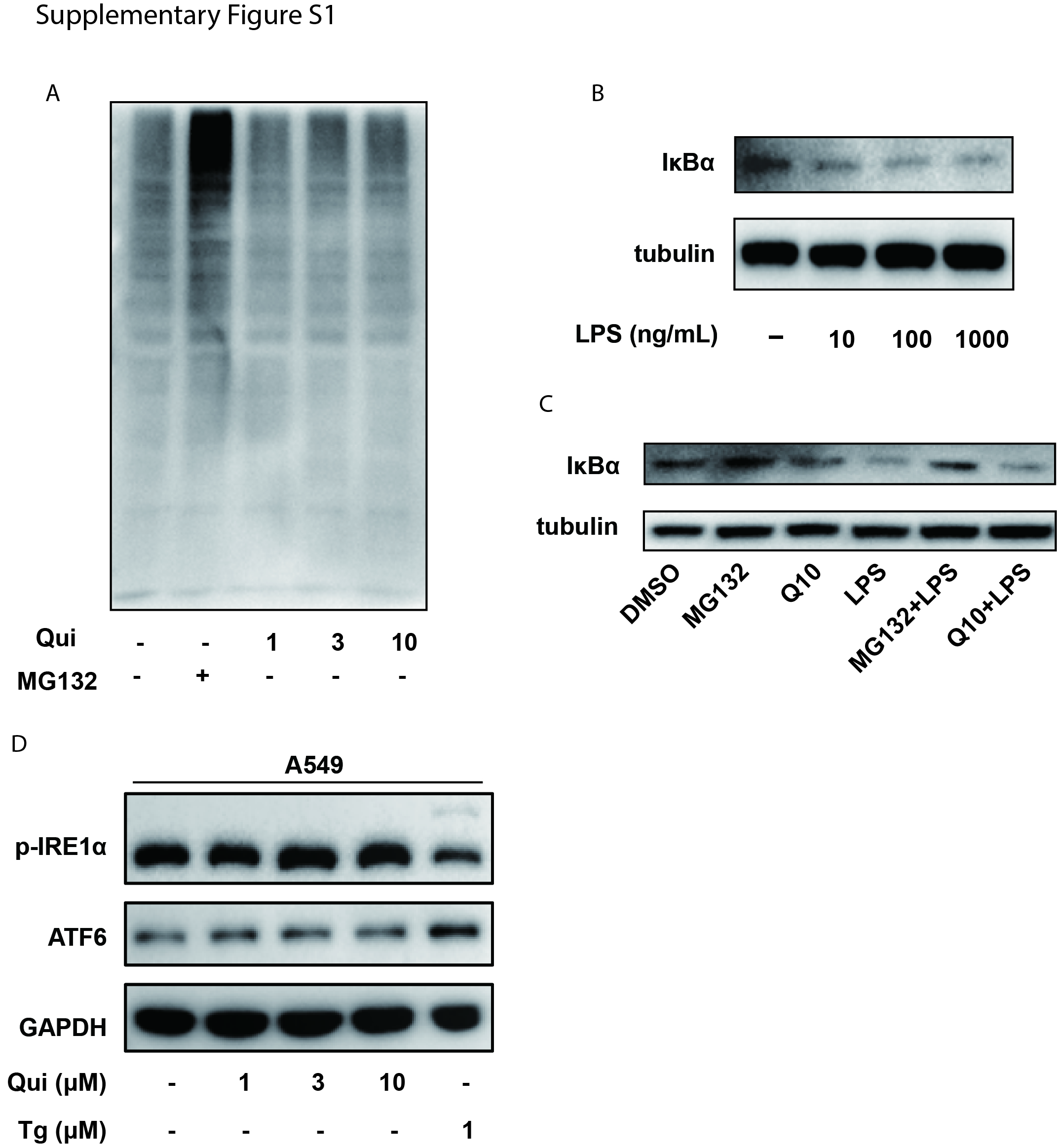

Supplement: Supplementary file 1 — Figure S1: The HDAC inhibitor quisinostat does not inhibit proteasomal activity. (A) NHK‐HeLa cells were treated with quisinostat (Qui) or MG132, and subjected to Western blot analysis for ubiquitination. (B‐C) NHK‐HeLa cells were treated with quisinostat and/or MG132, followed by LPS stimulation and subjected to Western blot analysis of IκBα degradation. (D) Increased concentrations of HDAC inhibitor quisinostat (Qui) does not activate IRE1α or AFT6 pathways of the ER stress response. [file JCMM-29-e70928-s001.jpg]

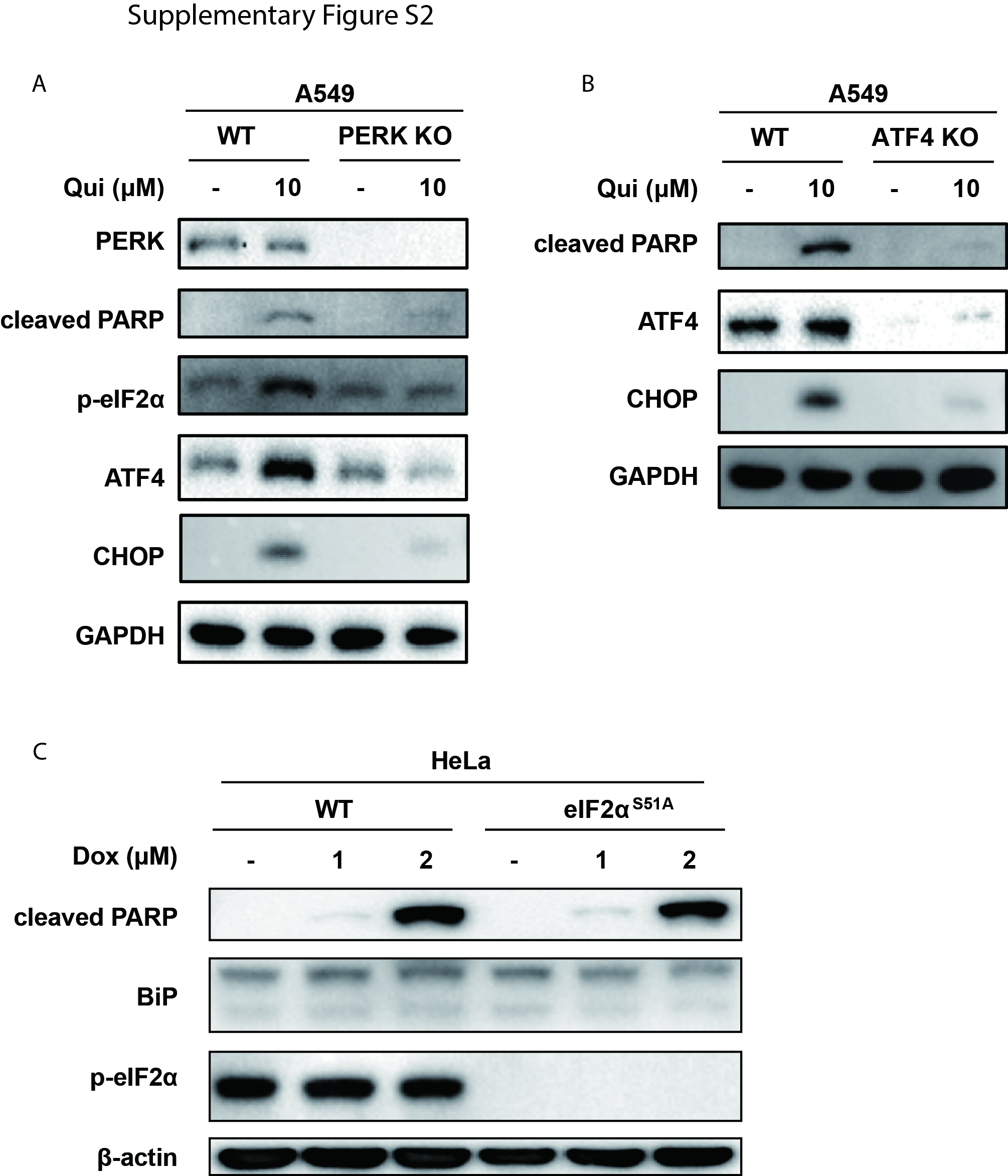

Supplement: Supplementary file 2 — Figure S2: The PERK‐eIF2α pathway is required for apoptosis induced by HDACi but not by doxorubicin (A) Knockout of PERK in A549 cells inhibits Qui‐induced apoptosis and p‐eIF2α, ATF4 and CHOP protein levels. (B) Knockout of ATF4 in A549 cells inhibits Qui‐induced apoptotic cell death. (C) Wildtype and eIF2αS51A‐mutant HeLa cells were treated with doxorubicin (Dox) for 24 h and subjected to Western blot analyses of cleaved PARP and BiP expressions, as well as eIF2α phosphorylation at Ser51. [file JCMM-29-e70928-s002.jpg]
